# New Design for Rapid Prototyping of Digital Master Casts for Multiple Dental Implant Restorations

**DOI:** 10.1371/journal.pone.0145253

**Published:** 2015-12-22

**Authors:** Luis Romero, Mariano Jiménez, María del Mar Espinosa, Manuel Domínguez

**Affiliations:** 1 Department of Construction and Fabrication Engineering, National Distance Education University (UNED), Madrid, Spain; 2 Department of Mechanical Engineering, Technical School of Engineering—ICAI, Comillas University, Madrid, Spain; Second University of Naples, ITALY

## Abstract

**Aim:**

This study proposes the replacement of all the physical devices used in the manufacturing of conventional prostheses through the use of digital tools, such as 3D scanners, CAD design software, 3D implants files, rapid prototyping machines or reverse engineering software, in order to develop laboratory work models from which to finish coatings for dental prostheses. Different types of dental prosthetic structures are used, which were adjusted by a non-rotatory threaded fixing system.

**Method:**

From a digital process, the relative positions of dental implants, soft tissue and adjacent teeth of edentulous or partially edentulous patients has been captured, and a maser working model which accurately replicates data relating to the patients oral cavity has been through treatment of three-dimensional digital data.

**Results:**

Compared with the conventional master cast, the results show a significant cost savings in attachments, as well as an increase in the quality of reproduction and accuracy of the master cast, with the consequent reduction in the number of patient consultation visits. The combination of software and hardware three-dimensional tools allows the optimization of the planning of dental implant-supported rehabilitations protocol, improving the predictability of clinical treatments and the production cost savings of master casts for restorations upon implants.

## Introduction

The traditional prosthesis fabrication process involves much handwork by dentists and dental technicians. The reason is that there was no available image data to construct computer aided design (CAD) models for practical prostheses, or a CAD model could not be constructed due to the complexity of the prosthesis. The fabrication of dental prostheses is also a problem. Practical dental restorations often have freeform surfaces (especially crowns). Bridges and implant structures tend to have features such as overhangs, undercuts, sharp corners, etc. [1].

Among the dental prostheses, crown fabrication occupies a large market share. Over 100 million crowns are made manually each year. The traditional crown fabrication process includes: tooth preparation (e.g. grinding), impression taking, treated tooth extraction, assembly of biting set, wax pattern making, centrifugal investment casting and finishing, and porcelain sintering or resin polymerization. All these steps depend significantly on the skill of the dental technician.

The most labor-intensive procedure lies in wax pattern making. The contour and fit of wax patterns is created by hand using small instruments and magnification. It takes a lot of time to transfer a pattern from a silicone impression to a wax pattern and then investment casting. A patient has to wait at least two weeks for a finished crown. Furthermore, the patient has no idea how the metallic crown looks and whether the crown satisfies his/her own esthetic considerations.

The dental prosthetic sector has evolved its manufacturing methods of dental prosthesis due the new technologies adapted to this sector by the software and hardware industrial manufacturers for designing and manufacturing of three-dimensional objects. More than a decade ago, the use of CAD/CAM systems and 3D scanners was exclusive of dental laboratories [2], by three-dimensional digitizing of master casts (model work in plaster that comes from the mold taken of the patient´s mouth, which replicates the hard and soft tissues of the oral cavity) in high precision 3D scanners. These are desktop scanners and they have a volume of approximately 90 x 90 x 90 mm scanning, so they are able to digitize the complete oral model.

The recent addition of the dental intraoral 3D scanners succeeds in creating three-dimensional images without taking alginate or silicone molds. To fully develop the dentures prosthesis, technicians need to have a physical model where they can position structures to perform esthetic completion of dental prostheses.

During the adapting process to the digital workflow, dental companies have been forced to develop software able to replicate the tooth model provenience of patient's three-dimensional mouth scan.

Models obtained by rapid prototyping have begun to play an important role in prosthetic procedures related with prosthetic restorations created by milling, casting and sintering processes.

Leading manufacturers of CAD systems for the dental sector have recently implanted among their design choices its proposal for a digital master model "model builder," which allows laboratory technicians to obtain a digital master model, e.g. Exocad, 3D-Shape and Dental Wings.

Software designs of models called model builder replicate the shape of the mouth in the form of organic geometry, which comes from the digitized plaster model (scanner) or from the mouth of the patient (intraoral scanner), displaying teeth, gingiva, tubers, etc. three-dimensionally.

Dental casts are widely used in dentistry to plan treatment and design prostheses. A digital dental cast can be saved along with patient data and retrieved conveniently when necessary. It can be transmitted over the network for remote diagnosis by a dentist. It can be used to measure distances and orientations of teeth with respect to each other. It also can be used to design restorations [3,4].

Prosthesis manufacturing directed toward esthetic and functional patient’s rehabilitation requires a master model that reproduces the maxillary morphology and its relationship with precision, as well as the exact position of the implants including implant replicas, their situation and the emergency shaft [5].

The diagnosis and rehabilitation process requires a proper planning and modeling of the study cast between the laboratory technician, the surgeon and the prosthodontist for the prosthetic study to generate correct impressions and appropriate intermaxillary records [6,7].

There are currently being developed new digital processes of oral implantology, based on the use of digital resources, both in the clinical phase and the prosthetic phase. They show that guided computer-assisted oral implantology is a viable implant technique when an adequate diagnosis and planning that addresses all of the treatment phases is made [8,9].

This study proposes obtaining a master cast using rapid prototyping of 3D models, previously obtained from the oral scanning generated data of an implant position, by using a measuring system with photogrammetry and a scanned plaster model.

The combination of both technologies [10,11] allows us to obtain soft and hard tissues of the patient with the implants positions relative to each other. This requires a treatment through CAD systems that make it possible to design and 3D printing of the master model using rapid prototyping techniques [12].

## Material and Methods

To avoid the errors generated in the process of creating the master cast with conventional techniques, and taking into account that the dental master cast is started by the medical dentist in the consultation, and is ended by the prosthetic technician in the laboratory, the ideal conditions to carry out a master cast are:

- Possibility to reproduce with high precision the oral cavity of the patient (mouth shape).
- Possibility to mount on articulator, to obtain the intermaxillary relationship.
- Possibility to structurally anchor prosthetic.
- Possibility to obtain accurately the angularities and distances between implant replicas.
- Possibility to replicate soft tissue that can be extracted.

To respond to these demands, this study presents a solution that allows the creation of a master dental cast in a digital way with as much or more fidelity than the one obtained with the traditional system, reducing the number of intermediate steps.

The process consists of several phases:

1. Digital measuring by non-contact photogrammetry of the implant positions to obtain the angularities and distances between the implants with accuracy;
2. Conventional alginate impression (faster and cheaper than silicones and polyethers) with healing pillars exposed to the view over the gingival;
3. Plaster emptying of alginate impression;
4. Plaster model 3D scan to obtain the virtual model;
5. Three-dimensional alignment of implant positions interrelated with respect to hard and soft tissues;
6. Information digital processing through the use of aided design and reverse engineering applications and creating the master cast (including gingival flange and subgingival emergence of implants).

To conduct this study, we have used an oral scanner, the reverse engineering software Geomagic Studio and the rapid prototyping technology based on resin injection and cured by UV light (IJP). These resources have been used for the development and application in the laboratory of the master cast in cases of prosthesis for totally edentulous and partially edentulous patients.

### Photogrammetry in digital scanning: reverse engineering

In contrast to the conventional impression techniques, the CAD technology uses another approach for data acquisition, namely digitizing. Review of literature showed that two different forms of digital data capturing systems are used in dental CAD technology: contact and non-contact digitizing methods [13].

Contact scanning procedures use a touch probe to record the relative position of points on the object’s surface. This procedure is commonly used in reverse engineering applications. The non-contact scanning procedure involves laser scanning (laser triangulation technology) and photogrammetry. Different types of dental structures need different types of scanning procedures in order to obtain a competitive complex 3D virtual model that can be used in CAD/CAM technologies [14].

Stereophotogrammetry is a system that uses two or more cameras to record several simultaneous photos from different viewpoints to obtain a digital 3D image [15].

**Fig 1:**
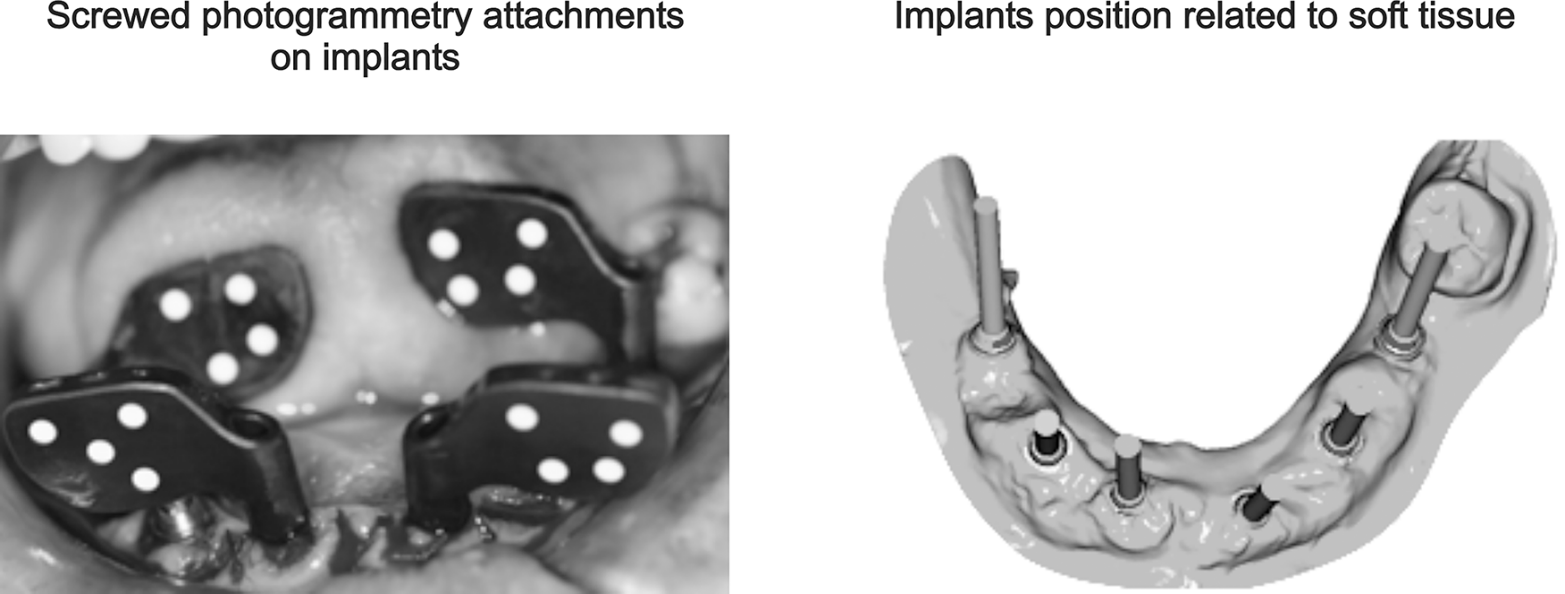
Use of an intraoral scanner and the digital master cast creation.

The main problem of the intraoral scanners currently used in clinics is due to the precision loss that is produced by the so-called "overlapping," which is a scanning alignment through common areas [10]. To achieve the precision required in multiple implant restorations, it is intended to use photogrammetry because this is the only technology that allows for accuracy of the required position (coordinates) of the implant (Fig 1).

Photogrammetry is the technology that all manufacturers of 3D scanners have adopted to carry out equipment calibration. Therefore, the 3D scanners’ accuracy depends on photogrammetry, technology which is considered as the most accurate in non-contact three-dimensional measurement [10].

The advantage of the methodology proposed in this study is based on the use of an intraoral scanner that uses photogrammetry, enabling scans of all patient implants without alignment by overlapping the various scans of each implant. Scanned alignments must be performed by a topology-based algorithm called "best fit." For this reason, intraoral scanner manufacturers do not offer rehabilitation of more than four teeth in the indications.

### Rapid prototyping technology to obtain the master cast

The potential advantages offered by the introduction of CAD in the field of removable partial denture framework design include automatic determination of a suggested path of insertion, instant elimination of unwanted undercuts and the equally rapid identification of useful undercuts, which are all crucial in dental technology. The potential advantages of a rapid manufacture approach are reduced manufacturing time, inherent repeatability and elimination of inter-operator variation [16,17].

Since inception, many rapid prototyping technologies have been employed for making medical prototypes in medicine and dentistry. Common technologies used in dentistry are stereolithography (SLA), inkjet-based system (3D printing—3DP), selective laser sintering (SLS) and fused deposition modeling (FDM). The materials that can be used are fairly diverse, but wax, plastics, ceramics and metals are all utilized by several teams for dental purposes [18].

Several methods can be employed to fabricate a physical prototype. These methods can be divided into two categories: subtractive and additive. They all start with a 3D CAD model of the anatomical area, which usually can be derived from X-ray CT or MRI data. The subtractive technique used is the conventional numerically controlled (NC) machining (milling).

The use of the rapid prototyping technology based on resin injection and cured by UV light (IJP) provides precision and materials combination that ensures the master model requirements are met [19]. In this study we have used the following materials and rapid prototyping technologies:

During the prosthetic phase, the laboratory technician uses the master cast to adjust, structure the wax and apply the porcelain according to routine laboratory procedures. In this last phase, the master model must undergo a heat team (pot) to polymerize the resin that surrounds the teeth and tooth structure.

Resin-curing temperature is 95°C during a time of 10–15 min. The mechanical and thermal characteristics of different prototyping materials to obtain the model are shown in Table 1 (all data provided by makers).

**Table 1:** Mechanical and thermal characteristics of different prototyping materials.

PROPERTY	NORM	PROTOTYPING TECHNOLOGIES–MATERIALS				
		OBJET (VeroDent)	OBJET (VeroDentPlus)	ENVISION (E-Denstone)	ProJET (Visijet e-Stone)	ProJET (Visijet PearlStone)
Tensile strength (Mpa)	ASTM D638-03 DIN EN ISO 527	50–60	54–65	56	37–39	40
Elasticity modulus (Mpa)	ASTM D638-04 DIN EN ISO 178	2000–3000	2200–3200	–	1500–1750	1794
Elongation at break (%)	ASTM D638-05 DIN EN ISO 527	10–25	15–25	3.5	10–23	17
Flexural strength (Mpa)	ASTM D790-03 DIN EN ISO 178	75–110	80–110	115	54–59	51
Flexural modulus (Mpa)	ASTM D790-04 DIN EN ISO 178	2200–3200	2400–3300	3350	1350–1750	1350–1750
Izod Notched Impact (J/m)	ASTM D256-06 ISO 180/1A	20–30	20–30	–	18–25	–
HDT (Heat Deflection Temperature) 0.45 MPa (°C)	D-648-06	45–50	45–50	–	58–63	–
HDT (Heat Deflection Temperature) 1.82 MPa (°C)	D-648-07	45–50	45–50	–	51–55	–
HDT (Heat Deflection Temperature) (°C)	ISO 11357-1/-3 DIN 53736	–	–	140	–	88
Water absorption (%)	D-570-98 24HR	1.1–1.5	1.2–1.5	–	–	
Glass Transition (Tg) (°C)	–	–	–	–	60	–

### Master cast created by 3D printing without analogs

**Fig 2:**
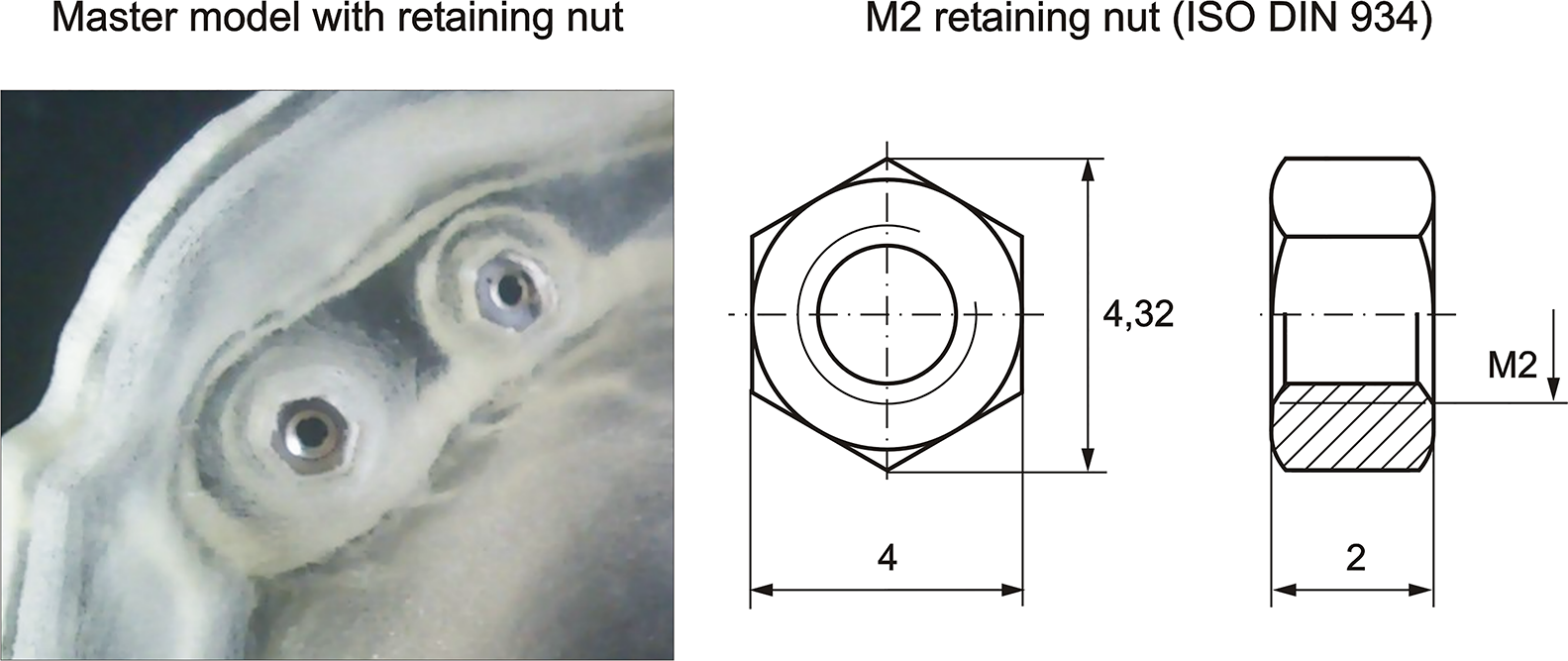
Master model with retaining nut.

In the design of the master cast presented, the inclusion of some holes located in the base of each implant is proposed, where tightly threaded nuts will allow adjustment, which will fix the implant screws and prevent rotation. M2 nuts (Fig 2) have been chosen.

The reason why these nuts are incorporated is that 3D printing still lacks (or the cost is too high) the detail of a threaded M2 fillet with sufficient quality and robust enough to withstand the tests to which the master cast must be submitted during the process.

## Results

### Digital master cast obtaining

**Fig 3:**
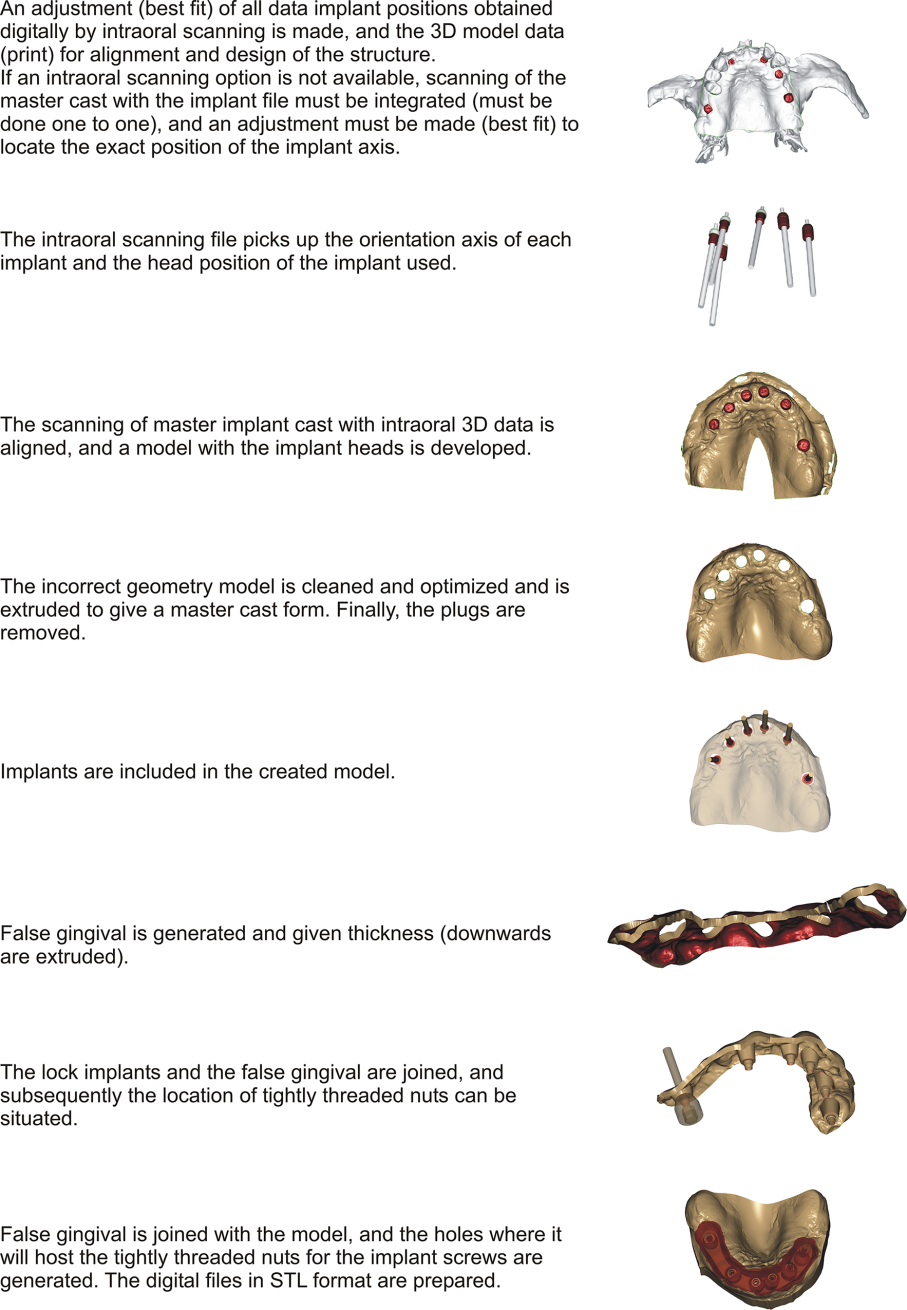
Digital process of master cast creation.

The process of creating the master cast without analogs from digitally data obtained by photogrammetry and subsequently treated with reverse engineering software is described in detail in Fig 3:

### Master cast created by 3D printing without analogs: master cast functionality

To manufacture the master cast without analogs the rapid prototyping technology based on resin injection and cured by UV light (IJP) is used, because it provides a perfect combination between the materials used and the precision obtained, which guarantees to fulfill all the necessary requirements for correct processing.

**Fig 4:**
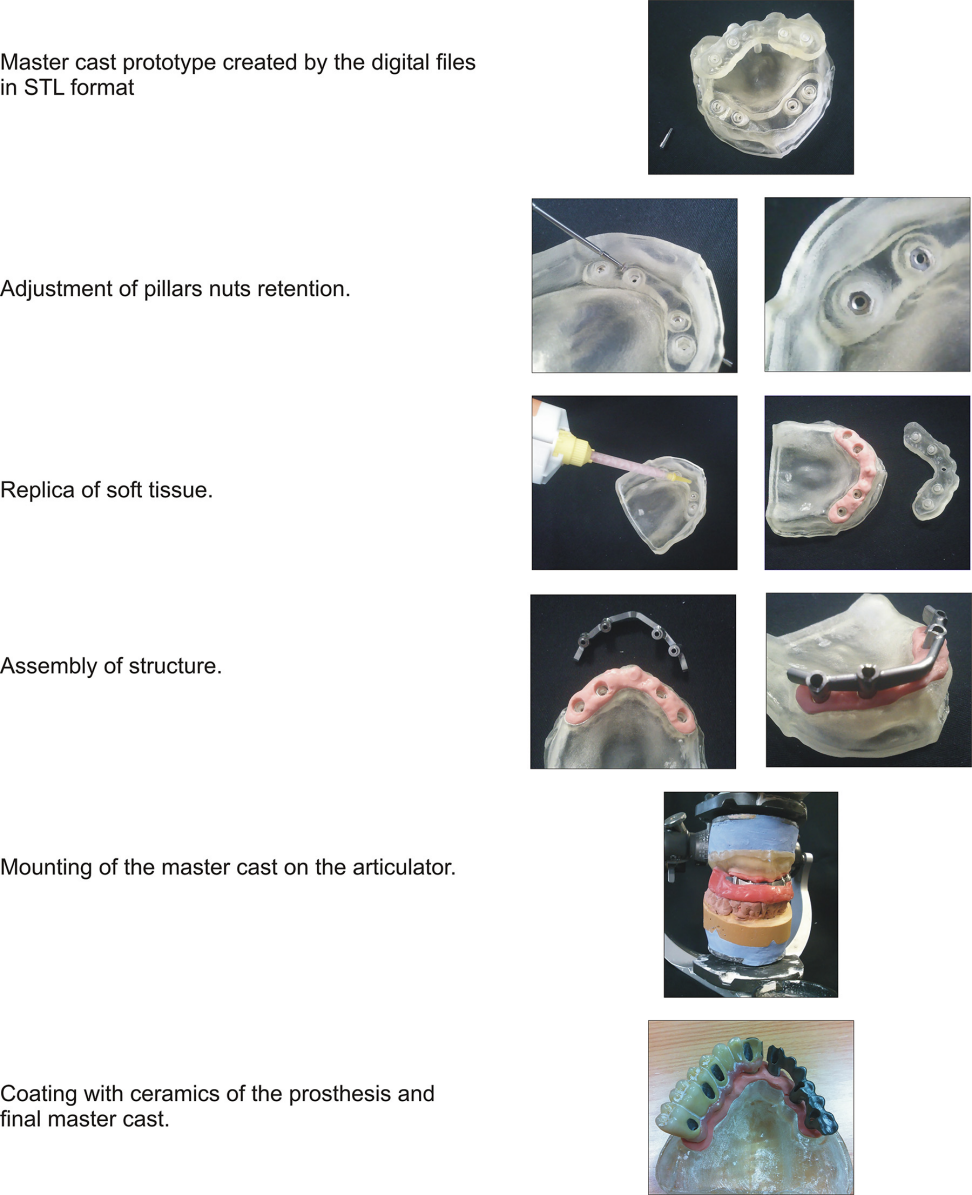
Functionality of prototyping master cast without analogs.

The digital process result described above is a master cast created using rapid prototyping technologies. This master cast obtains its functionality from the following preparation (Fig 4):

### Comparison in the master cast elaboration

Accuracy, times and operating costs spent to obtain the master cast for different prostheses, vary considerably according to the methodology used.

In order to clearly demonstrate the methodology advantages, a statistical comparison between conventional master cast, digital master cast with analogs and digital master cast without analogs is shown in Table 2. This comparison has been developed according to different tests realized to demonstrate the functionality of the master cast with different methodologies.

**Table 2:** Comparison between the methodologies for the master cast obtaining.

CRITERIA	Conventional master cast	Digital master cast with analogs	Digital master cast without analogs
Average time to obtain the final prosthesis	10–12 weeks	2–4 weeks	2–4 weeks
Total cost of the process	*****	****	***
Final accuracy of the prosthesis	0,1–1 mm	0,01–0,016 mm	0,01–0,016 mm
Master cast functionality	**	*****	*****
Master cast mounting on the articulator	****	****	****
Special prosthesis development	*	****	*****

As we can see in the table, the total cost to obtain the process in lower in digital methodology without analogs because impressions and casts are removed, as well as is the best way to obtain some prosthesis with special characteristics (for example, when there is no replica of the implant or when analogs are too close in the model).

The breakdown of the times and operating costs spent to obtain the master cast for a full denture depending on the tested methodologies is indicated in Table 3. As shown, the manufacture of the master cast using a digital process affects fewer activities in the laboratory and a reduction of overall times of the whole process. In addition the time employed for the final adjustment of the prosthesis in the mouth is much lower with digital methodology (in traditional methodology several visits of the patient are necessary for the prosthesis adjustment, while in digital way the adjustment is immediately on the first day).

**Table 3:** Analysis of times (min) and operating costs (€) in the master cast elaboration for a complete prosthesis.

**TRADITIONAL METHODOLOGY**			**DIGITAL METHODOLOGY**		
**PRE-SURGICAL PROTOCOL**					
**Operation**	**Time**	**Cost**	**Operation**	**Time**	**Cost**
Panoramic image (Ortopantomagrophy)	3	5	Panoramic image (Ortopantomagrophy)	3	5
Occlusal records and mouth models creation for surgical planning (guided or traditional surgery)	3	5	Occlusal records and mouth models creation for surgical planning (guided or traditional surgery)	3	5
**SURGICAL PROTOCOL I (AT CLINIC)**					
**Operation**	**Time**	**Cost**	**Operation**	**Time**	**Cost**
Impression post placement	4	3	Direct printing for implants and soft areas location	5	4
Impression	2	2	Impression post placement	4	3
–	–	–	Post position scanned	2	2
**PROSTHETIC PROCESS (AT LABORATORY)**					
**Operation**	**Time**	**Cost**	**Operation**	**Time**	**Cost**
Remove the impression post and relocation of analogs in the impression trace	5	8	Digital information processing: TAC, arcades impression and posts scanning	8	8
Placing replica material of the soft tissue around analogs joints	7	15	Master cast 3D design	5	5
Emptying the plaster impression and plaster separation	15	20	3D printing (rapid prototyping) of master cast	4	13
Unscrewed and removed from the posts of printing analogs	6	10	Preparation and placement the replica material of the soft tissue in the model	3	5
Empty the impression of the opposite arch	10	12	Pillar placement	5	3
Occlusal record realization for models articulation	12	15	Esthetic coating (porcelain)	7	10
Pillar placement	5	3	Finished off final prosthesis	4	2
Esthetic coating (porcelain)	7	10	-	-	-
Finished off final prosthesis	4	2	-	-	-
**SURGICAL PROTOCOL II (AT CLINIC)**					
**Operation**	**Time**	**Cost**	**Operation**	**Time**	**Cost**
Placement and fit of the prosthesis	7	10	Placement and fit of the prosthesis	7	10
**TRADITIONAL TOTAL**	**90**	**120**	**DIGITAL TOTAL**	**60**	**75**

### Demonstration of the master cast use

**Fig 5:**
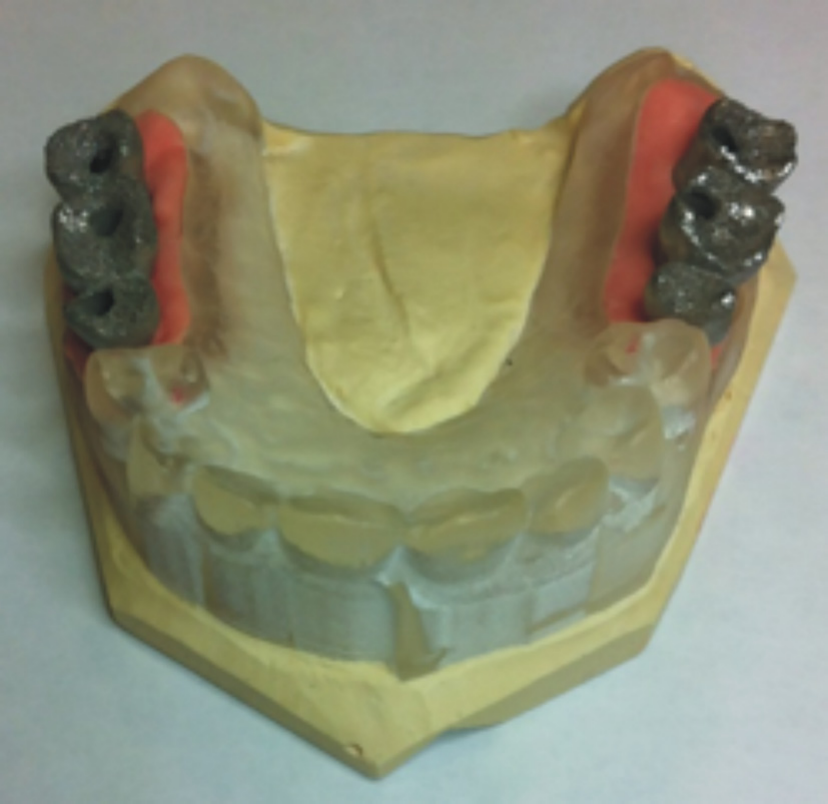
Master dental cast prototype with replica of the soft tissue and the steel structure of a partial prosthesis.

**Fig 6:**
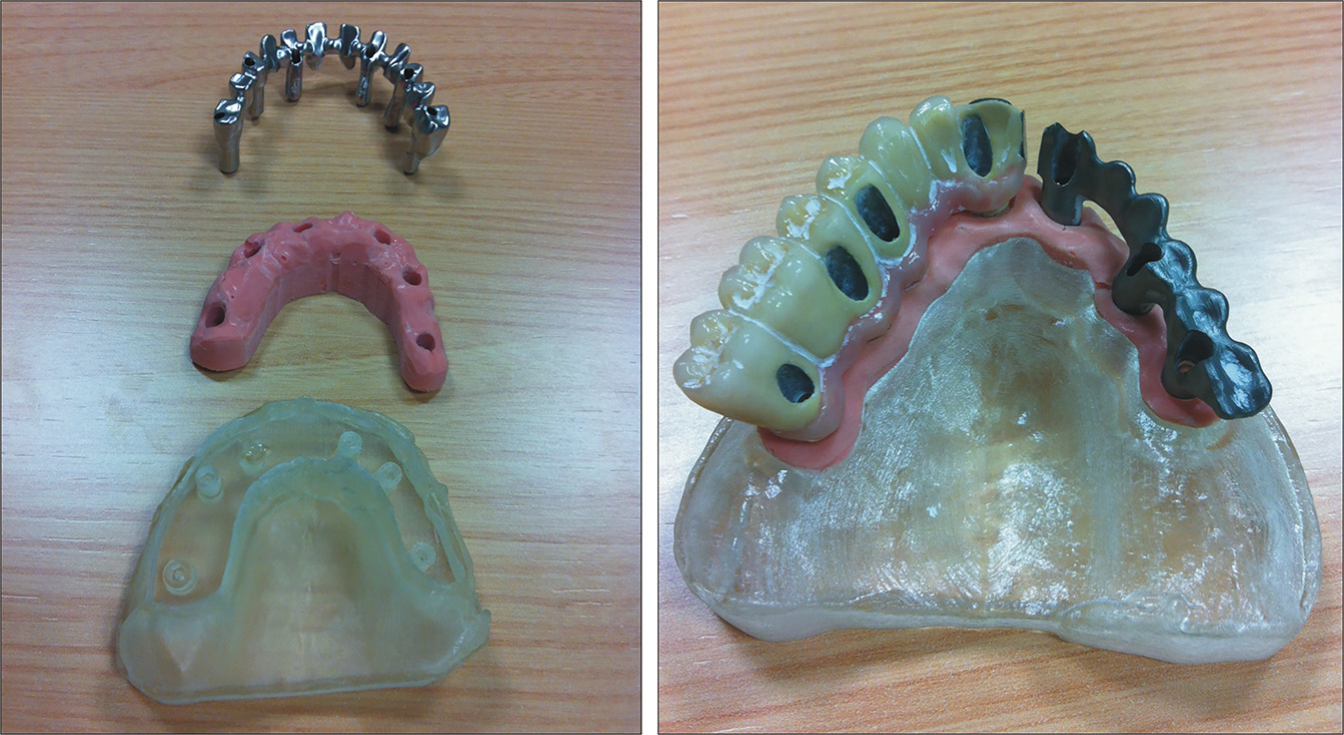
Master dental cast prototypes with replica of the soft tissue and the steel structure of a complete prosthesis.

In Figs 5 and 6, master dental cast prototypes are represented, with a replica of the soft tissue and the metallic structure (ceramic tile base) of a partial and a complete prosthesis, respectively.

Prosthetic structures built on a wrong model will not sit correctly on the implants, causing inadequate forces upon them; therefore, the use of appropriate materials significantly impacts the correct development of the model. In the tests, the prototyping materials of the model that have responded to the curing process demands have been VERODENT (OBJET) and Visijet Stone Fast (PROJET).

Mechanical and thermal characteristics of these materials provide to the master cast enough static rigidity which combined with the precision of the rapid prototyping technology based on resin injection and cured by UV light (IJP), allow to get a reliable master cast for the prosthetic process in the laboratory.

After the study of tests in edentulous and partially edentulous patients of different dental clinics, it is confirmed that the use of the master model built digitally (with the right materials) for the preparation of the different prosthesis types, ensures a decrease in costs and an increased precision in the mouth and, therefore, the patient’s satisfaction. In addition, the inclusion of some holes located in the base of each implant, will allow obtaining the master cast using 3D printing without the use of analogs, thus increasing the speed and accuracy and further reducing the overall costs of the process. Until this moment more than 25 tests for the use of the master cast mentioned have been realized. All tests were carried out in the clinic and the consent of all patients who participated was obtained (S1 Appendix).

## Discussion

Cemented prostheses on a natural tooth are fixed using between stump (carved tooth) and crown cementation. Due to this cementation space and inter-dental movement (which allows for the periodontal ligaments), accuracy is not a determining factor in these type of restorations [20].

However, the implant prosthesis cemented to intermediate pillars or screwed on requires great accuracy in terms of their spatial positions. If the relative positions between the implants do not match the relative positions of the structure, the settlement of this prosthetic on implants is not good. Therefore, when screwing the structure, tensile stress and bending forces are produced on dental implants and consequent transfer of tensional forces to the bony support of maxilla and mandible. It is demonstrated that the lack of fit between prosthesis and implants leads to loss of implants due to a lack of bone integration [21].

In addition the manufacturing of a master cast using conventional procedures requires a series of activities (impression, process, fit and mount on articulator) with a substantial cost and time requirement, as well as a few precision levels lower than those provided by digital procedures.

The master cast creation can be optimized in accuracy, time and cost through the use of digital implant methodology without analogs and rapid prototyping technologies. This master cast must reproduce precisely the maxillary morphology and its relationship as well as the exact position of the implants, including implant replicas, their situation and the emergency shaft.

The master cast is conditioned from the surgical phase by means of intraoral scanning of the impression posts position, which sets the precision and correct orientation of the implant through a scanner based on photogrammetry [10,14].

The methodology success consist in the use of the photogrammetry and the inclusion of holes located in the base of each implant where a tightly threaded M2 nut can be adjusted, fixing the implant screws and preventing its rotation, allows us to obtain the master cast using 3D printing without the use of analogs.

With conventional procedures, there are many intermediate steps for the preparation of the master cast from the start at a clinic until its completion in the laboratory. In all these steps, in which the human factor is involved, performance protocols are required for correct processing. The failures caused by the impressions on implants can be due to various reasons: bad settlement of the transfers on the implants, movement of the printing transfers, heterogeneous expansion of the plaster, incorrect grip to the printing replica due to lack of material. All these problems can be avoided by the use of the methodology proposed: during the prosthetic phase, a laboratory technician performs digital information processing and through the assisted design applications use obtains the master cast digitally, including gingival flange and the subgingival emergence of implants that enable an exact replica of the patient's mouth.

The use of the rapid prototyping technology based on resin injection and cured by UV light (IJP), seems the most suitable in the master cast manufacturing because it provides a perfect combination between the materials used and the precision obtained, which guarantees to fulfill all the necessary requirements for correct processing.

## Conclusions

The possibility of obtaining dental casts without analogs provides a faster, more accurate and, of course, a lower cost process. This issue has been raised in this study and has been resolved by obtaining a master cast by three-dimensional printing, in which the threading load is not supported by the base material but by a reusable nut, much more economical and reliable and which complies with the accuracy and repeatability requirements with equal or greater accuracy than traditional plaster master model.

This method results in a clear saving in direct costs because impressions and casts are removed, and physical attachments are not used because they are simulated through a digital library.

## Supporting Information

S1 Appendix Real cases of digital master casts for multiple dental implant restorations. (PDF)
