# Supplementary material for: New Design for Rapid Prototyping of Digital Master Casts for Multiple Dental Implant Restorations
Source: PLoS One. 2015 Dec 22;10(12):e0145253. doi: 10.1371/journal.pone.0145253 (PMC4687924; doi:10.1371/journal.pone.0145253)
Supplement: S1 Appendix — (PDF) [file pone.0145253.s001.pdf]

CAD/CAM QUALITY CONTROL

Madrid, June 30th, 2015

Adrián Hernández Gutiérrez
CEO - PIC Dental
Email: adrian.hernandez@picdental.com
T: +34 916 396 014
W: www.picdental.com

To Whom It May Concern:

Adrián Hernández Gutiérrez, as CEO of the IDITEC NORTH WEST S.L., I state that:

Researchers, L. Romero, M. Jimenez, M. M. Espinosa and M. Dominguez, authors of the article "New design for rapid prototyping of digital master casts for multiple dental implant restorations", have conducted in our Dental Prosthesis Laboratory, different tests to demonstrate the functionality of the digital master model in edentulous and partially edentulous patients of different dental clinics.

The tests have shown that the use of the master model built digitally (with the right materials) for the preparation of the different prostheses types, ensures a decrease in costs and an increased accuracy in the mouth and, therefore, the patient's and professionals satisfaction.

Below are pictures of some of the tests performed. Currently, the digital model proposed research, is being used in our laboratory as a reference master model. During 2014 more than 80 clincial cases were undertaken successfully with these types of master models and approved by dental technicians and clinics.

Should you need any further information about these tests, please feel free to contact me.

CAD/CAM QUALITY CONTROL
www.picdental.com

Adrián Hernández Gutiérrez
CEO PIC Dental

REAL CASES OF DIGITAL MASTER CASTS FOR MULTIPLE DENTAL IMPLANT RESTORATIONS

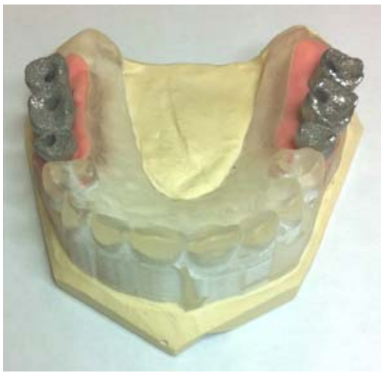

Test nº 1. Master cast for chrome cobalt implant bridges

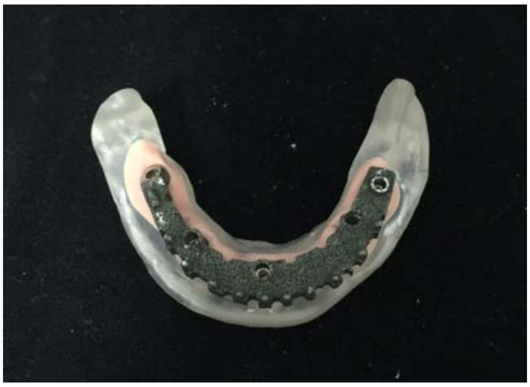

Test nº 2. Master cast for screw-retained hybrid bar

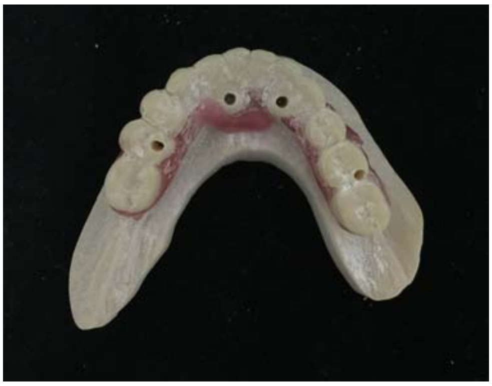

Test nº 3. Master cast for provisional rapid prototyping moke-up

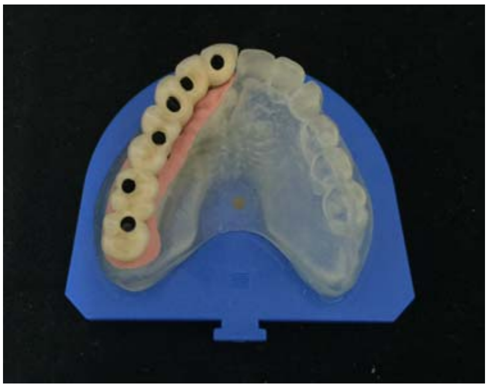

Test nº 4. Master cast for seven units metal ceramic bridge

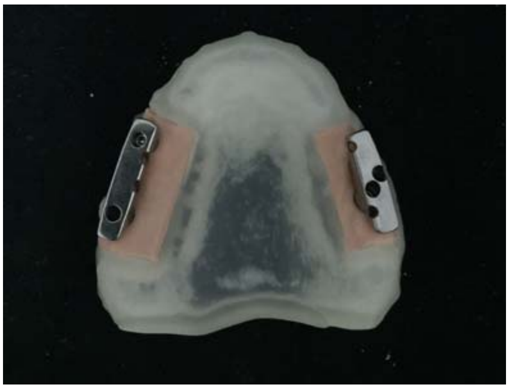

Test nº 5. Master cast for titanium bars for an overdenture

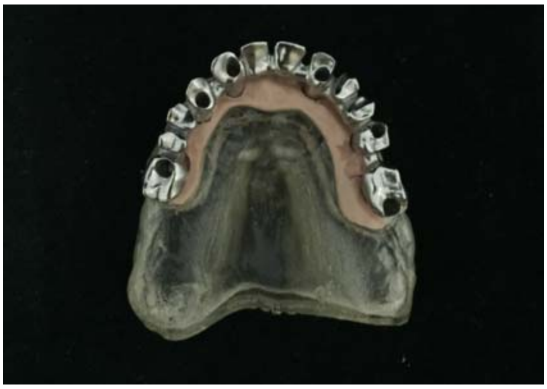

Test nº 6. Master cast for milled chrome cobalt full arch structure

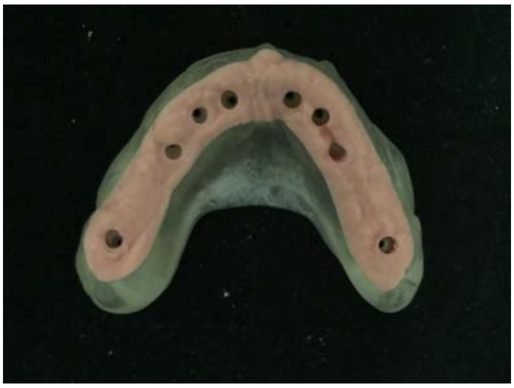

Test nº 7. Master cast for false gingiva material detailed

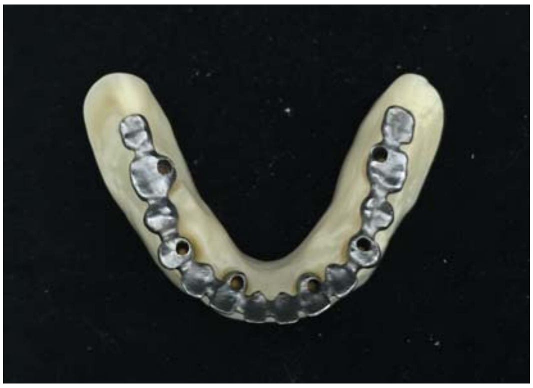

Test nº 8. Master cast for laser sintering chrome cobalt of full arch structure

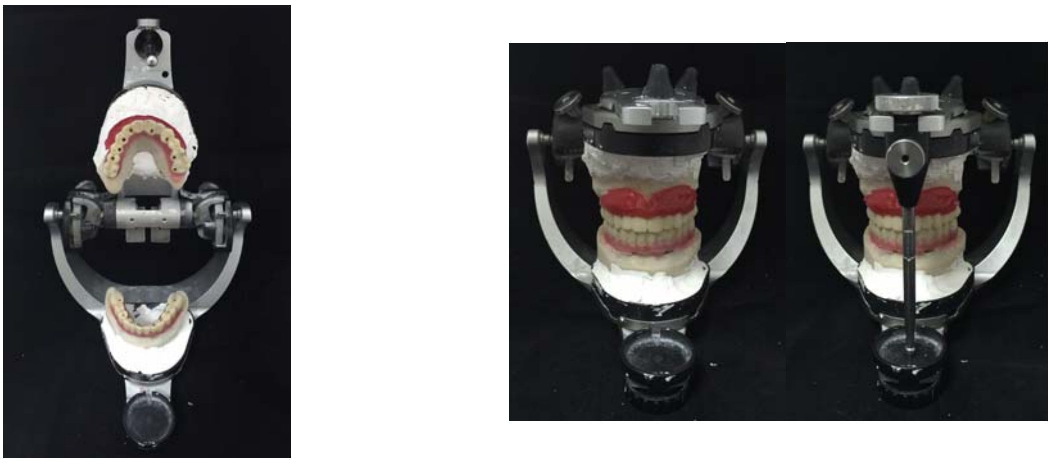

Test nº 9. Master cast assembly on a semi-adjustable articulator for fixed screw-retained restorations (TMJ movements simulation)

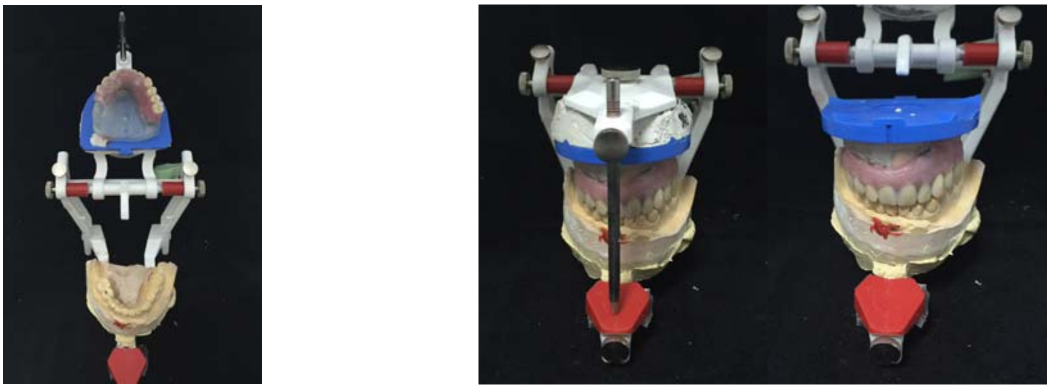

Test nº 10. Master cast assembly on a non adjustable articulator for fixed screw-retained restorations (open-close bite simulation)
